# Development of a questionnaire measuring instrumental activities of daily living (IADL) in patients with brain tumors: a pilot study

**DOI:** 10.1007/s11060-016-2352-1

**Published:** 2017-02-01

**Authors:** Q. Oort, L. Dirven, W. Meijer, S. A. M. Sikkes, B. M. J. Uitdehaag, J. C. Reijneveld, M. J. B. Taphoorn

**Affiliations:** 1 Department of Neurology and Brain Tumor Center Amsterdam, VU University Medical Center, PO Box 7057, 1007 MB Amsterdam, The Netherlands; 2 Department of Neurology, Leiden University Medical Center, Leiden, The Netherlands; 3 Department of Epidemiology and Biostatistics, VU University Medical Center, Amsterdam, The Netherlands; 4 Alzheimer Center, VU University Medical Center, Amsterdam, The Netherlands; 5 Department of Neurology, Academic Medical Center, Amsterdam, The Netherlands; 6 Department of Neurology, Medical Center Haaglanden, The Hague, The Netherlands

**Keywords:** IADL, Activities of daily living, Glioma, Brain tumor, Daily functioning, Amsterdam IADL Questionnaire

## Abstract

Both dementia and brain tumor patients exhibit cognitive decline during the course of their disease. They might therefore experience similar problems with cognitively complex daily activities (i.e., instrumental activities of daily living (IADL)). The study's objective is to evaluate if the Amsterdam IADL Questionnaire^©^ (A-IADL-Q), a 70-item IADL questionnaire developed for and validated in early dementia patients, is also applicable to glioma patients. The evaluation consisted of three steps. Predetermined decision rules defined which activities were retained, altered, added or excluded. In the first step, 6 neuro-oncology health care professionals (HCP) and 10 glioma patient-proxy dyads were asked to evaluate the 70 A-IADL-Q activities. In the second step, in-depth interviews were conducted with 6 HCPs and 6 other patient-proxy dyads to generate relevant activities specific to glioma patients not covered by the A-IADL-Q. In the third step, 6 new patient-proxy dyads were cognitively debriefed with the list of activities constructed in the previous steps. Results indicated that in step 1, after alterations and exclusions, 28/70 activities could be retained. Nine newly generated activities were subsequently added in step 2. In step 3, the 37 activities were presented to the patient-proxy dyads. Based on their input, several additional alterations and exclusions were made resulting in a list of 32 activities. In conclusion, this evaluation of the A-IADL-Q showed that dementia-specific IADL activities are only partly applicable to glioma patients, and that the addition of glioma specific IADL activities is necessary to capture the IADL construct. This underlines the need for a disease-specific IADL questionnaire for brain tumor patients.

## Introduction

Traditional outcome measures used in clinical trials with brain tumor patients are overall and progression-free survival and tumor response on magnetic resonance imaging. In addition, information on patients’ functioning and well-being has become increasingly important, especially in patients with an incurable disease. The quality of survival in brain tumor patients is arguably at least as important as the duration of survival [1].

One way to measure patients’ functioning is with activities of daily living (ADL). ADL can be divided into two categories, basic activities of daily living (BADL) and instrumental activities of daily living (IADL). BADL are basic skills needed for self-maintenance such as feeding, bathing, dressing, and toileting [2]. IADL on the other hand, includes more cognitively complex activities such as food preparation, handling finances, shopping, housekeeping or using electronics (i.e., a telephone or computer) [3]. These capacities are essential to function autonomously within society. IADL functions seem to be sensitive to the early effects of cognitive decline [4]. Since cognitive decline is characteristic of brain tumor patients, measuring IADL is especially relevant.

Commonly used ADL scales and questionnaires in brain tumor patients such as the Barthel Index [5] and the Karnofsky performance status [6], only cover BADL. Even though the functional independence measure (FIM) [7], whether or not in combination with the functional assessment measure (FAM) [8], assesses problems in activities of daily living, it also includes cognitive abilities, such as concentration and memory, and emotional status. These items cover different constructs. Moreover, these questionnaires have several other limitations. First of all, most of these questionnaires were developed in the late 1950s, with more recent revisions in the early 1990s. Advances in technology have changed our daily environment dramatically (e.g., the use of mobile phones, computers and household appliances) [9]. Secondly, the quality demands on self-report questionnaires have increased in the meantime. Although most psychometric properties of these questionnaires have been shown to be adequate [10, 11], there are still some limitations. For example, the FIM-FAM has not been validated in brain tumor patients.

Although there is no gold standard to measure IADL specifically in brain tumor patients, a recently proxy-based questionnaire was developed and validated to measure IADL problems in patients with early dementia [12]. Since cognitive deficits are present in both early dementia patients and brain tumor patients during the course of the disease, regardless of whether this is a result of the disease or its treatment, we expected they might exhibit similar IADL problems.

Therefore, the objective of this pilot study was to evaluate if the Amsterdam IADL Questionnaire^©^ (A-IADL-Q) is also applicable to glioma patients.

## Methods

### Participants

Adult patients (1) with a histologically confirmed diffuse glioma (World Health Organisation (WHO) grade II diffuse astrocytoma, oligodendroglioma or oligoastrocytoma, WHO grade III anaplastic astrocytoma, anaplastic oligodendroglioma or anaplastic oligoastrocytoma or WHO grade IV glioblastoma), (2) who are in frequent contact (daily or weekly) with their proxies and (3) native Dutch, were eligible and recruited from the neuro-oncology outpatient clinics of the VU University Medical Center in Amsterdam and the Medical Center Haaglanden in The Hague, the Netherlands. Before each outpatient clinic, all attending patients were screened for eligibility and those eligible were invited to participate. With this method, we hoped to ensure a representative sample of the diffuse glioma population. Furthermore, two neuro-oncologists, two specialised neuro-oncology research nurses and two neuropsychologists were included as health care professionals (HCPs). The study was approved by the Medical Ethical Committee of the VU University Medical Center, Amsterdam, The Netherlands.

### Materials

The Amsterdam IADL Questionnaire^©^ (A-IADL-Q) is an informant-based questionnaire [4]. The A-IADL-Q has been shown to have favourable psychometric properties, high internal consistency and test–retest reliability, and good content and construct validity in memory clinic patients [4, 12]. The A-IADL-Q consists of 70 items and is computerized and adaptive, and scores are calculated using item response theory (IRT; statistical framework that describes the relationship between a patient’s response to an item and his/her level of the underlying construct, e.g., IADL, that is being measured). For each activity, the ‘main’ item would be whether or not the person had done the activity in the past 4 weeks, and the ‘follow-up’ item would be either; whether or not they had experienced more difficulties with the activity *or* for what reason they were unable to do the activity (due to physical issues, mental issues, never partaken in the activity or other). The A-IADL-Q was found to be relevant and important by both patients and proxies, the respondent burden to be limited, and the method of data collection (on a tablet) to be user-friendly [12].

### Procedure

Using a multi-step approach, the applicability of the A-IADL-Q for glioma patients was evaluated [4, 13].

#### Step 1: Evaluation of the activities in the Amsterdam IADL Questionnaire^©^

Six HCPs as well as ten patients with a glioma and their proxies (total n = 2 × 10 = 20) were requested to evaluate the main A-IADL-Q activities. HCPs evaluated the activities on three aspects; (I) can the activity be considered IADL using the proposed definition (=‘IADL are complex activities with little automated skills for which multiple cognitive processes are necessary’ [4]), (II) is the activity likely to be affected in glioma patients, and (III) is the item clearly defined and formulated? The inclusion criteria were as follows; ≥5/6 HCPs had to affirm that the activity was IADL, ≥4/6 HCPs had to recognize the activity as likely to be affected and ≥5/6 HCPs had to consider the item to be clearly formulated to be considered clear.

Patients and their proxies only needed to consider two questions; (I) is the activity likely to be affected in glioma patients, and (II) is the item clearly defined and formulated? At least 3/10 patients/proxies had to recognize the activity as likely to be affected and ≥9/10 patients/proxies had to consider the item to be clearly formulated to be considered clear.

#### Step 2: Generating relevant IADL activities not covered by the Amsterdam IADL Questionnaire^©^

The same HCPs (n = 6) and another group of patients and their proxies (total n = 2 × 6 = 12) were interviewed to assess which IADL are affected in glioma patients, but not covered by the existing activities in the A-IADL-Q. To do so, in-depth semi-structured interviews with open questions were conducted using the ‘sampling-to-redundancy’ criterion [14]. Only the activities that were in accordance with the proposed IADL definition and mentioned by ≥3 HCPs or patients and proxies were included.

The A-IADL-Q activities retained after step 1 were activities that were considered either (a) IADL, affected and clearly defined *or* (b) IADL and affected, but not clearly defined. In the latter case, the items were rephrased. In addition, if step 2 revealed that certain relevant activities were not covered by the A-IADL-Q, an item on this activity was formulated. These two steps resulted in a new set of activities that was believed to measure IADL in glioma patients, and was cognitively debriefed in step 3.

#### Step 3: Cognitive debriefing of the new list of activities

A new group of 6 glioma patients and their proxies (total n = 2 × 6 = 12) underwent a cognitive debriefing. This technique was used to test whether all activities were interpreted as intended. If ≥2 participants indicated similar mentions of ambiguity or repetitiveness, we rephrased, merged or omitted the item.

### Statistics

Descriptive statistics were used to analyse the clinical and demographic variables of the study population and the responses of the participants. Statistical analyses were performed using SPSS version 21.0 software (SPSS, Chicago, IL, USA).

## Results

A total of 50 participants were included in this study; 22 patients, 22 proxies and 6 HCPs (Table 1). Ten low-grade and 12 high-grade glioma patients were included. More than half of the patients were male. All proxies were the patient’s partner. Most proxies have been in a relationship with the patient for over 10 years. The six experienced HCPs consisted of 2 neuro-oncologists, 2 specialised neuro-oncology research nurses and 2 neuropsychologists.

**Table 1:** Participants’ clinical and demographic characteristics

Participants	Patients (N = 22)	Proxies (N = 22)	HCPs (N = 6)
**Gender** (male), n (%)	12 (55%)	10 (45%)	2 (33%)
**Age**, mean (SD)	52 (13)	53 (14)	47 (12)
Median (range)	50 (25–76)	51 (27–75)	47 (−64)
**Level of education** ^a^, n (%)			
0–4	13 (59%)	13 (59%)	
5–8	9 (41%)	9 (41%)	
**Relation to the patient**, n (%)			
Partner		22 (100%)	
**Contact intensity**, n (%)			
Living together		22 (100%)	
**Duration of the relationship (in years)**, mean (SD)		23 (11.87)	
<5 years		0 (0%)	
5–10 years		6 (27%)	
>10 years		16 (73%)	
**Histological diagnosis**, n (%)			
WHO grade II	10 (46%)		
WHO grade III	6 (27%)		
WHO grade IV	6 (27%)		
**Disease status**, n (%)			
Stable	19 (86%)		
Progressive	3 (14%)		
**Anti-tumor treatment status**, n (%)			
Current anti-tumor treatment	4 (18%)		
No current anti-tumor treatment	18 (82%)		

^a^ *Level of education* according to international standard classification of education ranging from 0 (low) to 8 (high) [15]

### Step 1: Evaluation of the activities in the Amsterdam IADL Questionnaire^©^

#### Question I: Can the activity be considered IADL?

Six HCPs evaluated if the 70 activities in the A-IADL-Q could be considered as IADL. Almost all items were considered to be IADL (67/70; 96%). The disagreement in these three cases can be explained by the format in which the items were presented (adaptive vs. non-adaptive). Although the activities were considered as IADL, the 70 main items in the A-IADL-Q were not formulated in a way that they could be considered IADL, while the follow-up item was. The A-IADL-Q follow-up items were in line with the suggestions made by our HCPs.

#### Question II: Affected in glioma patients?

Ten patients, ten proxies and six HCPs evaluated if the 70 activities in the A-IADL-Q were affected in glioma patients. HCPs considered 44/70 (63%) of the activities as likely to be affected in glioma patients. Patients and proxies recognized only 14/70 (20%) and 15/70 (21%) of the activities as affected, respectively. Concordance between patients and proxies was 97%.

#### Question III: Clearly defined and formulated?

Participants evaluated if the 70 items in the A-IADL-Q were clearly defined and formulated. HCPs rated only half (36/70, 51%) of the items as clearly defined and formulated. On the other hand, patients and proxies rated 65/70 (93%) and 60/70 (82%) items as clear, respectively. Thirty-three out of 70 (47%) items were considered clearly formulated by all participants.

**Fig. 1:**
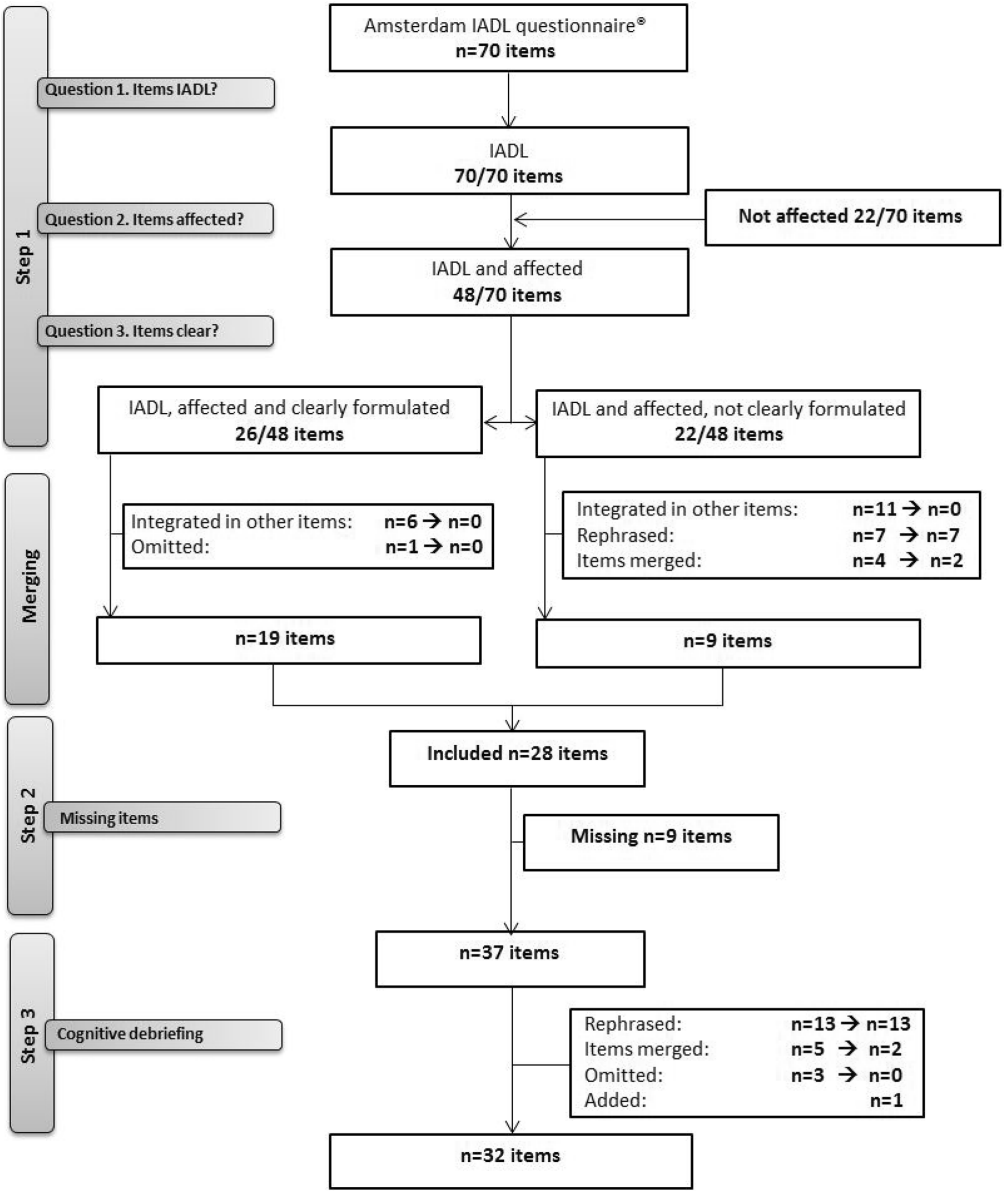
Flowchart reflecting the steps taken to assess the applicability of the Amsterdam IADL Questionnaire^©^ for glioma patients

Based on the input of all participants, 48/70 (69%) activities were considered likely to be affected in glioma patients by either the patients, proxies or HCPs, with 22/48 items being considered both affected and clear (Fig. 1). Of the 26/48 unclear items, seven items were rephrased, four items were merged and 11 items were integrated in other items. In some cases (n = 7), items were considered to be clear, but HCPs suggested the items should be omitted because they were considered repetitive (n = 6) or redundant (n = 1). Step 1 resulted in a list of 28 items being considered both IADL, affected and clearly formulated.

### Step 2: Generating relevant IADL activities not covered by the Amsterdam IADL Questionnaire^©^

The results of the in-depth interviews revealed several IADL which are affected in glioma patients, but not covered by the A-IADL-Q (Table 2). During the interviews, both HCPs and the patients and proxies mentioned ‘multitasking’ and ‘keeping appointments’ as missing. Moreover, HCPs mentioned numerous other activities affected in glioma patients, such as ‘organizing activities’. The activity uniquely mentioned by the patients and their proxies was, ‘having a conversation with several people at the same time’. Nine new activities were formulated.

**Table 2:** Novel instrumental activities of daily living generated by ≥ 3 HCPs (n = 6) and ≥ 3 patient-proxy dyads (n = 6) during the in-depth interviews

Newly generated activities	HCPs n = 6	Patients and proxies n = 6
Organizing/initiating social activities	6 (100%)	–
Reading a book or newspaper	6 (100%)	–
Multitasking	5 (83%)	4 (67%)
Finishing work on time	5 (83%)	–
Following a TV programme or movie	5 (83%)	–
Keeping appointments	4 (67%)	4 (67%)
Having a conversation with several people at the same time	–	4 (67%)
Learning new things	3 (50%)	–
Overseeing one’s own activities	3 (50%)	–

### Step 3: Cognitive debriefing of the new list of activities

The 28 activities resulting from step 1 and the nine newly formulated activities in step 2 were merged (Fig. 1), resulting in a provisional item list of 37 activities (Appendix 1), which was cognitively debriefed by six new patient-proxy dyads. Based on their input several additional alterations and exclusions were made (Fig. 1), resulting in a final item list consisting of 32 activities (Appendix 2).

## Discussion

Based on the similarities in cognitive decline between patients with early dementia and a glioma, we hypothesized that the A-IADL-Q, developed for and validated in dementia patients, could possibly be applicable for glioma patients. Surprisingly, results showed that, even though eventually all activities of the A-IADL-Q were considered IADL, only a number of the activities were considered applicable to glioma patients. Furthermore, several relevant IADL to glioma patients were not covered by the questionnaire.

A striking but explicable result was the discrepancy between the HCPs and the patients and proxies. HCPs considered about three times as many activities affected in glioma patients compared to patients and proxies. Indeed, patients and proxies only have their own situation as reference, whereas HCPs have broader knowledge of and experience with potential problems glioma patients might face. On the other hand, patients and proxies considered almost twice as many items as well-defined compared to HCPs. This also is understandable considering the HCPs have a more critical approach to evaluating items than patients and proxies. For instance, HCPs evaluated the wording more closely to ensure it is appropriate for people with different levels of education and background. However, in most cases the HCPs in this study seemed to rate the items as unclearly defined because of the redundancy and repetitiveness of the items. This is most likely due to the fact that the A-IADL-Q is scored using IRT and in this study all main items were presented.

The A-IADL-Q might not be as applicable to glioma patients for several reasons. First of all, factors such as age might account for the discrepancies. Although the risk of a glioma increases with age, many relatively young individuals are diagnosed with this disease when compared to dementia. Our study population was about 10 years younger than the study population used in the development of the A-IADL-Q. Especially the types of activities generated in step 2 (activities focus mainly on socially active and occupationally related subjects) seem to point to a difference in generation or stage of life. Another factor might be a difference in disease severity. Early dementia patients group might already be more severely cognitive impaired when seeking medical assistance. Moreover, other cognitive domains might be affected in glioma patients. The most prominent impairment in patients with dementia is memory loss [16]. Glioma patients can exhibit a range of neurocognitive deficits in areas such as language, memory, executive functioning, attention and motor function [17–19]. Tucha and colleagues documented the incidence of cognitive impairments immediately after diagnosis but before the start of treatment among patients with frontal or temporal brain tumors and found that ±90% of patients demonstrated impairments in at least one aspect of cognition and one-third of the patients demonstrated impaired functioning in eight or more cognitive areas [18]. The most commonly identified impairment in the study was in executive functioning. A number of studies have shown a strong association between executive functioning and having difficulties with appropriately initiating and completing IADL [3]. This suggests that the discrepancies between dementia patients and brain tumor patients might be due to differences in the predominant underlying cognitive impairment and the number of impaired cognitive domains. Moreover, cognitive problems in glioma patients might also be more diversified due to differences in tumor characteristics (e.g., volume and location) within the patient group. Another aspect might be that brain tumor patients could also have problems with IADL due to physical problems (for instance loss of motor function or sensory problems), epilepsy or non-tumor related issues such as advanced age, comorbidity and legal issues (e.g., not allowed to drive a car because of epilepsy) [20, 21].

Evidently, this study has some limitations. First of all, the sample size was relatively small. Therefore, the problems the patients indicated may not completely reflect those of the ‘whole’ glioma population. Secondly, a selection bias could have been introduced by including the more healthy patients in this study [22]; patients with, for example, severe cognitive impairments, psychological distress or declined physical conditions often not participate [20]. Indeed, the majority of the patients in this study had stable disease. We have tried to overcome this by also consulting HCPs selected from different disciplines (physicians, psychologist and nurses), who encounter patients from all stages of the disease. Furthermore, the generalizability of this study is limited because only Dutch natives and only glioma patients were included. Patients with other types of primary brain tumors or those with brain metastases were not included. Finally, the newly generated activities have not been validated as IADL by HCPs.

## Future perspective

The results of this pilot study clearly suggest the necessity of a disease-specific IADL questionnaire for brain tumor patients. Currently, the development of an IADL questionnaire for brain tumor patients is in progress, in accordance with the EORTC Quality of Life Group guidelines for developing questionnaire modules [13]. The study will include a larger patient population, with both patients with a primary brain tumor and patients with brain metastases, and the questionnaire will be cross-culturally validated. The list of 32 activities derived from this pilot study will be used in the developmental process of the questionnaire.

## Appendix 1: IADL item list after step 1 and 2

During the past month, did you have difficulty with…

**Household duties:**
1. Grocery shopping independently?
2. Buying the correct groceries?
3. Preparing sandwich meals?
4. Preparing/cooking hot meals?
5. Using domestic appliances (like the microwave, oven, dishwasher, coffee maker or washing machine)?
6. Doing chores or minor repairs in or around the house?

**Finances and administration:**

(7) Doing the financial administration?
(8) Using electronic banking?
(9) Withdrawing money at an ATM or paying with a card in a store?
(10) Paying cash?
(11) Filling in forms?

**Appliances:**

(12) Using a mobile telephone or smartphone?
(13) Understanding an instruction manual?
(14) Operating the television remote control?

**Work:**

(15) Functioning adequately (as previously) at work?
(16) Finishing your work on time?

**Appointments:**

(17) Making appointments independently?
(18) Keeping your appointments?
(19) Planning or organizing an activity?

**Computer:**

(20) Using a computer?
(21) Searching the Internet for information?
(22) Using email?

**Social activities:**

(23) Having a conversation with several people?

**Transport:**

(24) Driving a car?
(25) Driving a car safely?
(26) Operating a car navigation system?
(27) Using public transport independently?
(28) Finding your way in an unfamiliar surrounding?

**Leisure time:**

(29) Following programs on television?
(30) Reading a book, magazine or newspaper?
(31) Going on holiday or a day out?

**General:**

(32) Finding important items (such as wallet, keys or phone) around the house?
(33) Dealing with unexpected circumstances?
(34) Overseeing your activities?
(35) Doing several things at once (multitasking)?
(36) Being able to take your medication independently?
(37) Learning to do new things?

## Appendix 2: IADL item list after step 3

During the past month, did you have difficulty with…  

**Household duties:**
1. Grocery shopping independently?
2. Preparing a meal?
3. Using household appliances (such as the telephone, microwave, oven, dishwasher, coffee maker or washing machine)?
4. Doing chores or minor repairs in or around the house?

**Finances and administration:**

(5) Keeping the financial administration in order?
(6) Using electronic banking?
(7) Withdrawing money at an ATM or paying with a card in a store?
(8) Paying cash?
(9) Filling in (digital) forms?

**Appliances:**

(10) Using a mobile telephone or smartphone?
(11) Following an instruction manual?
(12) Using a (television) remote control?
(13) Using a computer (like browsing, emailing or using computer programs)?

**Work:**

(14) Functioning normally (as previously) at work?
(15) Finishing your work on time?

**Appointments:**

(16) Making appointments independently?
(17) Keeping appointments?
(18) Planning or organizing an activity (such as a birthday, dinner or a night out)?

**Social activities:**

(19) Having a one-on-one conversation?
(20) Having a group discussion?

**Transport:**

(21) Driving a car safely?
(22) Using a navigation system?
(23) Using public transport independently?
(24) Finding the way in unfamiliar surrounding?

**Leisure time:**

(25) Following a television program or movie?
(26) (Comprehensively) reading a book, magazine or newspaper?

**General:**

(27) Finding important items (such as wallet, keys or phone) around the house?
(28) Changing your activity when an unexpected situation arises?
(29) Overseeing your activities?
(30) Doing several things at once (multitasking)?
(31) Managing your own medication independently?
(32) Learning to do new things (such as a course, computer program or appliance)?
